# High Genetic Diversity Despite the Potential for Stepping-Stone Colonizations in an Invasive Species of Gecko on Moorea, French Polynesia

**DOI:** 10.1371/journal.pone.0026874

**Published:** 2011-11-02

**Authors:** Maria A. Tonione, Natalie Reeder, Craig C. Moritz

**Affiliations:** 1 Museum of Vertebrate Zoology, University of California, Berkeley, California, United States of America; 2 Department of Biology, San Francisco State University, San Francisco, California, United States of America; University of Otago, New Zealand

## Abstract

Invasive species often have reduced genetic diversity, but the opposite can be true if there have been multiple introductions and genetic admixture. Reduced diversity is most likely soon after establishment, in remote locations, when there is lower propagule pressure and with stepping-stone colonizations. The common house gecko (*Hemidactylus frenatus*) was introduced to Moorea, French Polynesia in the remote eastern Pacific within the last two decades and accordingly is expected to exhibit low diversity. In contrast, we show that *H. frenatus* on Moorea has exceptionally high genetic diversity, similar to that near the native range in Asia and much higher than reported for other Pacific island reptiles. The high diversity in this recently founded population likely reflects extensive genetic admixture in source population(s) and a life history that promotes retention of diversity. These observations point to the importance of understanding range-wide dynamics of genetic admixture in highly invasive species.

## Introduction

Among the most important causes of native-species extinctions is interaction with invasive species [1]. Considerable effort has been expended on genetic analyses of invasive species to infer invasion history, the dynamics of genetic diversity, and how this might contribute to potential to adapt to and thrive within non-native habitats [2]–[Bibr pone.0026874-Dlugosch1] [4]. On one hand, recently introduced populations might have reduced diversity due to founder events [5]–[Bibr pone.0026874-Tsutsui1] [7], in which case their evident ability to adapt and spread represents something of a paradox [8]. Alternatively, multiple introductions involving divergent source populations can increase genetic diversity in invasive populations and promote evolutionary flexibility and the rate of spread [9]–[Bibr pone.0026874-Lucek1] [Bibr pone.0026874-Zalewski1] [Bibr pone.0026874-Verhoeven1] [13]. The latter outcome depends on high propagule pressure (i.e. geographic proximity to diverse source populations and/or location on transport routes; [14]) and often takes time to develop [3], [9]. The former outcome, reduced diversity, is more probable in recently colonized and geographically remote locations, especially if there have been sequential founder events associated with a stepping-stone colonization process (e.g. [15]–[Bibr pone.0026874-Clegg1] [Bibr pone.0026874-Thulin1] [18]).

The islands of French Polynesia are among the most remote in the world and have a disharmonic biota derived by a mix of natural stepping-stone colonization from the western Pacific, natural colonizations from the east, and subsequent radiations, all overlain by recent human introductions [19]. The depauperate reptile fauna is dominated by species of geckos (including parthenogenetic species) and skinks, which colonized naturally (e.g. possibly *Gehyra oceanica*, [20]) or as commensals in association with Polynesian migration [21]. A characteristic feature of these lizards, associated with evolutionarily recent and presumably stepping-stone colonizations, is genetic uniformity from the islands of the western Pacific to French Polynesia [20], [22]–[Bibr pone.0026874-Bruna1] [24]. Human-mediated introductions continue, the most recent invader, the Gold Dust Day Gecko (*Phelsuma laticauda*), being recorded on the island for the first time in 2006 [25].

The focus of the present study, the Common House Gecko *Hemidactylus frenatus*, is a prime example of an invasive species with demonstrated effects on native reptile faunas [26]–[Bibr pone.0026874-Cole1] [28]. The native range of *H. frenatus* is uncertain. Recent studies have suggested a south Asian origin, although it may be that Indian and Sri Lankan lineages represent a different species from that in Myanmar and eastwards, as well as the invasive range of *H. frenatus* [29], [30]. Wherever in south Asia it originated, *H. frenatus* is now circumtropical, mostly as a human commensal, on both continents and islands. Invasion of the western tropical Pacific was assumed to have commenced some 4000 years ago with the early Polynesian and Melanesian migration, and continued with *H. frenatus* spreading as a stowaway on shipping and cargo boats [26], [28]. Since the mid 20^th^ century, *H. frenatus* has spread through the eastern Pacific beginning in Hawaii in 1951, then Fiji in 1960 and Vanuatu in the 1970s–1980s [26]. Across the tropical Pacific, *H. frenatus* is largely restricted to human structures [26], [28], but has also been found in low numbers invading natural habitat as well (e.g. [27], [28], [31]). *H. frenatus* was first reported in French Polynesia in 1989 [21] and was not present on the adjacent island of Moorea in late 1980's (C. Moritz, pers. obs., R. Fisher pers. comm.; [32]). From this we can conclude *H. frenatus* has been on Moorea for at most 22 years (∼20 generations).

Given the evident recent colonization, geographic remoteness, the likelihood of stepping-stone introductions, and also the low diversity observed in other species of lizards from the region, we expected *H. frenatus* on Moorea to have low genetic diversity, which could restrict their capacity to adapt to non-commensal environments. Alternatively, as preliminary analyses of this species on western Pacific islands revealed unexpectedly high mtDNA diversity [24], it is possible that the recently established Moorea population could have substantial diversity as a consequence of originating from an already admixed invasive population, although even here we would expect somewhat lower diversity due to sequential founder events. We test these alternative hypotheses by comparing genetic diversity on Moorea to that in south-east Asia (Myanmar and Indonesia), in or near the presumed native range. We also document the spread of *H. frenatus* on Moorea.

## Results

### Distribution on Moorea

**Figure 1:**
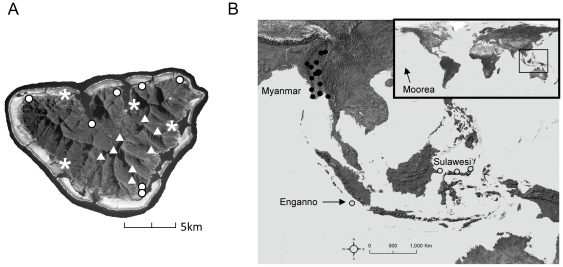
Sampled localities in Southeast Asia and on Moorea. (A) Map of Moorea showing *H. frenatus* localities sampled (open circles), localities *H. frenatus* was seen but not sampled (asterisks) and localities no *H. frenatus* was found (triangles). (B) Sampled localities in Southeast Asia. Black circles represent individuals from Myanmar; gray circles represent individuals from Indonesia. Inset: Map of the world; arrow pointing to approximate locality of Moorea.

Since its arrival no more than 22 years ago, *H. frenatus* has dispersed to occupy dwellings and other man-made structures in high numbers around the entire perimeter of the island; in contrast, no individuals of this highly conspicuous species were detected in repeated surveys of the interior forest (Fig. 1A). *Lepidodactylus lugubris*, previously common on buildings [32], and Gehyra oceanica common in interior forest but also found on buildings, are now rare relative to *H. frenatus* (Table S1).

### Mitochondrial DNA

**Figure 2:**
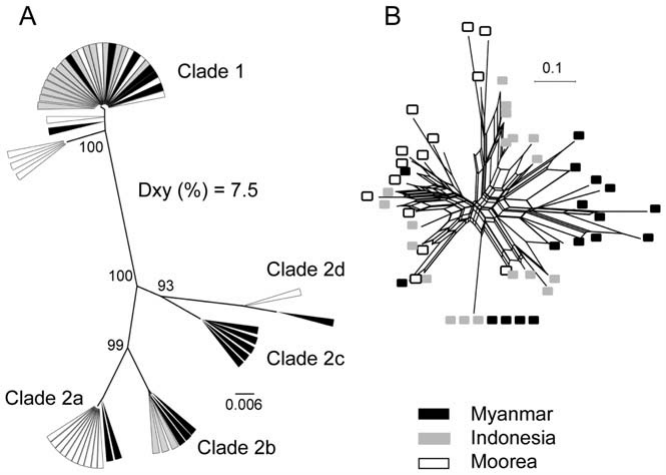
Phylogenetic relationships inferred from maximum likelihood analysis of CO1 and phylogenetic network of nuclear loci. (A) Unrooted ML network of CO1. Numbers on the branches represent ML bootstrap values. Geographic locality is represented by rectangle color as in figure 1B. (B) Phylogenetic networks of the concatenated nuclear loci using uncorrected ‘p’ genetic distances, colored by geographic locality.

*H. frenatus* from Moorea (n = 21 samples) has highly diverse mtDNA, comparable to the variation observed across south-east Asia. There are 2 major groups (group 1 and group 2; Fig. 2A) and can be further divided into 4 subgroups (group 2a, 2b, 2c, 2d; Fig. 2A). The recently invaded Moorea population has three highly divergent haplotypes (group 1, 2a, 2d; Fig. 2A), each of which is closely related to those from Myanmar (Fig. 2A, mean *Dxy* among groups (%) = 7.5). Samples from the Indonesian islands, closer to the presumed native mainland range, are represented in each of the two major mtDNA groups, and again are closely related to haplotypes from Myanmar (Fig. 2A). The diversity within Moorea (θ_π_ = 0.043±0.022) is similar to that across all of Myanmar (θ_π_ = 0.038±0.019) and greater than found in our geographically dispersed samples in Indonesia (θ_π_ = 0.019±0.010; Table 1). Though not significant, estimates of Tajima's D for both Myanmar and Moorea were positive (1.14 and 1.86 respectively), i.e. in the direction expected with recent admixture. Curiously, despite high overall diversity within Myanmar, there is little obvious phylogeographic structure within this region, with the two major mtDNA groups having broad and overlapping distributions (Fig. S1). Net sequence divergences (*Da*) between regions were relatively low, ranging from 0.39–1.24% (Table 1). Samples from Moorea and Myanmar showed no significant genetic divergence, whereas those from the Indonesian sample were significantly divergent from both Myanmar and Moorea (*F*_ST_ = 0.27 and 0.25 respectively, p<0.001; Table 1).

**Table 1:** Population diversity^1^.

	mtDNA				nDNA			
	n	Moorea	Indonesia	Myanmar	n	Moorea	Indonesia	Myanmar
Moorea	22	0.04	1.13	0.39	18	0.02	0.0019	0.0049
Indonesia	18	**0.25**	0.02	1.24	18	**0.11**	0.01	0.0034
Myanmar	17	0.08	**0.27**	0.05	17	**0.20**	**0.16**	0.02

1 Based on Tamura-Nei corrected average pairwise divergence (*D*a; above diagonal); within-population θ_π_ (diagonal); population pairwise *F*_ST_ based on Tamura & Nei genetic differences (below diagonal). Bold numbers indicate statistically significant (p<0.05).

To enable comparison with published data from India and elsewhere, we sequenced representatives of each major lineage for the *12S rDNA* gene. The major groups observed across Myanmar, Indonesia, and Moorea cluster with those from elsewhere in the introduced range (Columbia, Hawaii, Papua New Guinea, and China; [33]–[Bibr pone.0026874-Whiting1] [35]), and are highly divergent from the Indian haplotypes ([29], [30]; Fig. S2).

### Nuclear DNA

We obtained sequence data for three nuclear loci from nearly all individuals and, again, the Moorea population had high diversity (Figs. 2B, S3; Table 1). As for mtDNA, multi-locus nDNA sequence diversity within Moorea (θ_π_ = 0.016±0.008) is on par with that across Myanmar (θ_π_ = 0.022±0.01) and higher than that in Indonesia (0.014±0.007). Population differentiation at the nuclear level was significant (p<0.001) for every pair of populations by country (Table 1; for information on individual nuclear loci see Table S2 and Fig. S3). However, assignment of individuals to genetic groups using Structure did not reveal any coherent population structure at either K = 2 or K = 3 (Fig. S4). As a preliminary test for cyto-nuclear disequilibrium, we also tested for nDNA divergence between the two major mtDNA groups in both Myanmar and Moorea; neither test was significant (nDNA *F*_ST_ = 0.0628, −0.0918 respectively, p>0.05).

## Discussion

Despite being geographically remote and having the potential for stepping-stone introductions, the *H. frenatus* population on Moorea has high genetic diversity, similar to that across widely separated samples from in or near to the native range (Myanmar) and higher than that across Indonesia, a colonized area much closer to the mainland. The high mtDNA diversity of *H. frenatus* on Moorea contrasts strongly with low diversity observed in other lizards that have colonized the eastern Pacific islands [20], [22]–[24].

It has been hypothesized that geckos experience faster mitochondrial evolution [36]–[Bibr pone.0026874-Jesus1] [38] in particular when comparisons to nuclear diversity can be made [39], [40]. Multilocus analyses of intraspecific gekkonid lineages in their native range typically reveal several-fold lower diversity at nuclear loci than for mtDNA (e.g. *Heteronotia binoei*; average nDNA/mtDNA π = 0.11; [41]) as expected given the higher mutation rate for mtDNA. However, in other studies of geckos following human-mediated introductions, there is unexpected high nuclear diversity compared to mitochondrial diversity perhaps as a result of a selective sweep reducing the latter (average nDNA/mtDNA π = 0.84; [42], [43]). A higher than usual ratio of nDNA/mtDNA diversity in each of the three regional samples of *H. frenatus* (nDNA/mtDNA π; Moorea = 0.38, Myanmar = 0.46, Indonesia = 0.73) was also found in this study but more sampling of non-admixed populations within the native range are needed to understand this pattern.

The genetic admixture in the Moorea population of *H. frenatus* can be explained by multiple introductions from geographically/genetically distinct native-range sources (*in situ* admixture, e.g. [9]) or by one or more introductions from already genetically admixed populations(s), most likely from elsewhere in the Pacific (ancestral admixture). Multiple secondary introductions from already admixed populations could also cause this increase in diversity and appear to be the case here. These introductions likely represent an ongoing chain of admixture starting in or near the native range, as divergent groups within Myanmar are neither geographically structured (Fig. S1) nor distinct at nuclear loci. Overall, the evidence points to recent admixture among genetically divergent populations as the source of the high genetic diversity of the Moorea population of *H. frenatus*.

Based on limited geographic and genetic sampling, the Pacific Ocean populations are genetically more similar to those from Myanmar and other introduced populations (e.g. China, Hawaii; [34], [35]) than those in the presumed South-Asian native range (India, see Figure S2). Coordinated genetic analyses across the mainland Asian range of *H. frenatus* and related species are necessary to clarify species borders and relationships [37], [44] and to identify source populations (e.g. [9]), assuming these are not already obscured by secondary introductions.

It is clear that the large and genetically diverse introduced population on Moorea avoided a genetic bottleneck, and the presence of high genetic diversity could have contributed to invasion success (e.g. [3]). But high diversity at neutral markers is not a prerequisite for invasive success; there have been many successful invasions with reduced genetic diversity (e.g. [3], [6], [45], [46]). In addition, the range of *H. frenatus* has recently expanded to include the western coast of Madagascar even though this population appears to have low genetic diversity for mtDNA relative to those studied here [47]. Life history characteristics can play an important role in promoting high retention of genetic diversity. For example, *H. frenatus* females are able to store enough sperm to form six clutches across the span of a year [48]; thus, even a small number of inseminated females can co-found a new population with high genetic diversity. In addition, *H. frenatus* eggs are often found under palm shingles of native houses or bark and when washed out to sea avoid desiccation [49].

Reptile extinctions on islands are causing significant decline in biodiversity [27], [50], [51]. The spread of invasive reptiles such as *H. frenatus* will only exacerbate the problem as many studies on *H. frenatus* have shown that this gecko is a strong competitor and often displaces native geckos [26], [27], [52], [53]. So far, no *H. frenatus* have been found in the interior forest of Moorea (Fig. 1A) though expansion from commensal into natural habitats has been observed elsewhere [28]. Climatic niche modeling of *H. frenatus* predicts an even further expansion of suitable habitat particularly in South America and Africa [54].

Both genetic and natural history traits appear to be important factors leading to *H. frenatus*' successful colonization of the Pacific Islands. It is unclear which factors weigh most heavily in allowing introduced species to establish and spread, but it seems necessary to consider that founder populations may, through several mechanisms, maintain more genetic diversity than previously thought. Future work with *H. frenatus* would be to investigate the native range and the routes of introductions to test alternative invasion scenarios and estimate demographic parameters relevant to introductions and ultimately test other ecological or evolutionary hypotheses underlying invasions [55].

## Materials and Methods

### Surveys and Sampling

General sampling for *H. frenatus* and other geckos on Moorea was done for approximately one week in March 2006, one week in August 2008, and one week in April 2010. These included repeated surveys of human dwellings around the perimeter of the island, and primarily forest habitats in the interior. During the first trip in March 2006, we conducted a survey March 28^th^, from approximately 2100 h to 2300 h. During this informal survey, we drove around the perimeter of the island and stopped at seven different buildings and counted the gecko species we found (Table S1). All research was conducted under UC Berkeley Animal Care and Use Committee approval #R278-0509 (permit information: Protocol D'accueil D'un Chercheur ou Enseignant-Chercheur etranger, Delegation a la Recherche (De la Polynesie Francaise)).

### Molecular

We extracted DNA from vouchered specimens at the Museum of Vertebrate Zoology, University of California, Berkeley, and the California Academy of Science (Table S3). Twenty one sequences were from specimens sampled on Moorea while thirty-six samples were from within or adjacent to the native range including Myanmar and Indonesia (Figs. 1A, 1B). Total genomic DNA was isolated from liver using a standard high salt extraction [56]. We amplified approximately 650 basepairs of the mitochondrial cytochrome *c* oxidase subunit I (CO1). To compare our major mtDNA lineages with those published previously, we chose a representative from each group and sequenced *12S rDNA* ([29], [33]–[Bibr pone.0026874-Whiting1] [35]; Fig. S2). We also PCR amplified three nuclear autosomal loci for a total of 762 bases (Table 2). Nuclear autosomal marker rpl14 was previously published in Fujita et al. [41]. The two other nuclear markers (LFABP and rpl18) were developed with the same methods as Fujita et al. [41] by random sequencing from a cDNA library for *Heteronotia binoei*, another closely related gekkonine lizard. Primers and conditions for both mitochondrial and nuclear markers can be found in Table 2. We amplified genomic DNA in 12.5 µL reactions with an initial denaturation at 94°C for 3 min, followed by 35 cycles of denaturation at 94°C for 30 s, 30 s annealing at specific temperatures for each locus as outlined in Table 2, and extension at 72°C for 60 s. Each reaction had ∼20 ng genomic DNA, 0.5 µM of each primer, 1× PCR Buffer, 1.2 mM MgCl_2_, 0.6 mM dNTPs, and 0.5 U of Taq. We then purified the product using ExoSAP-IT (USB) and sequenced using BigDye v3.1 (Applied Biosystems). The purified sequence product was sequenced on an ABI 3730 automated DNA sequencer. Sequences were aligned using Geneious Pro (Biomatters, Auckland, New Zealand).

**Table 2:** Marker conditions used in this study.

Gene	Size (bp)	TA (°C)	Primers (5′-3′)	Source
CO1	650	48	dgLCO-1490: GGTCAACAAATCATAAAGAYATYGG	Meyer [73]
			dgHCO-2198: TAAACTTCAGGGTGACCAAARAAYCA	
*rpl14*	271	62	exon 1: ACTGGTAGCAATTGTGGATGTTATCG	Fujita et al. [41]
			exon 2: GAACTTGAGAACGAAGTCAGTCAGTTG	
*rpl18*	226	60	n108: GTTGTCTTGAAGCAGGAATCAACTTC	This paper
			n109: ATTCATAAATTCTTCGCAGCTCCTTC	
*LFABP*	265	65	n32: AGGTGTATGTCCAGGAGAATTATGAAG	This paper
			n33: GGATTTCAATGGTCGGCTTAATATC	

### Mitochondrial DNA

Phylogenetic analyses for CO1 using maximum likelihood (ML) were conducted in RaxML [57] and partitioned by codon position. ML nodal support was calculated by analyzing 100 bootstrap replicates. To estimate the extent and distribution of sequence diversity, populations were grouped by collection country and compared using Arlequin v3.1 [58]. For each population pair we estimated divergence among populations (*D*xy and *D*a using the Tamura-Nei model; [59]) and pairwise *F*_ST_ based on Tamura & Nei's model of sequence evolution [60]. Additionally, we estimated within-population diversity (θ_π_; [61]) and Tajima's D [62] for each population.

For the 12 s phylogeny, we conducted a Bayesian phylogenetic analysis using MrBayes v 3.1.2 [63]. We first implemented MrModelTest v2.3 [64] to establish the HKY+1+G model best representative of the substitution process for the dataset. We allowed four incrementally heated Markov chains to proceed for 20 million generations, sampling every 1000 generations. Bayesian posterior probabilities were estimated after 2000 samples were discarded for burn-in. The tree was rooted with *H. brookii* from Carrazan and Arnold (Fig. S2; [33]).

### Nuclear DNA

We inferred haplotypes computationally using the program PHASE v.2.1. [65], [66]. We then used these phased genotypes coded as allelic states to infer population structure in our nuclear loci using the Bayesian assignment Structure v2.3.1 [67]. We ran 10 iterations for K = 2 and K = 3 (Fig. S4). We used a burn-in of 100,000 steps, MCMC length of 500,000 steps and the correlated allele frequencies and the admixture ancestry model.

A multilocus genetic network was used in this study to visualize individual relationships. Using this approach, we are able to show a more realistic visual representation of ambiguous or incompatible phylogenetic signals, such as one that might be seen with recombination, using a net-like scheme [68]. In order to construct this genetic network, we first calculated the genetic distances among individuals at each nuclear locus. We used the uncorrected ‘p’ genetic distances and the HKY85 model [69] in PAUP v4.0b10 [70]. The program POFAD v1.03 [71] was used to combine the genetic distance matrices of the three nuclear loci. We visualized this network using the Neighbor Net algorithm [72] in SplitsTree v4.6 [68]. Individual nuclear neighbor-joining trees can be found in Figure S3 and diversity at each locus in Table S2. As for mitochondrial DNA, we used Arlequin to estimate *D*a, θ_π_, and *F*_ST_ across nuclear loci.

## Supporting Information

Figure S1 **Map of Myanmar showing location of major CO1 lineages.**
(TIF)

Figure S2 **Bayesian phylogeny of 12S sequences.** Neighbor joining tree includes 12S sequences from this study, Bansal and Karanth [29], Carranza and Arnold [33], Feng et al. [35], and Whiting et al. [34]. Numbers to the right of the names represent major CO1 groups found in this study. Posterior probabilities for the major groups are included on the branches.
(TIF)

Figure S3 **Unrooted Neighbor-joining network of (A) rpl18, (B) rpl14, and (C) LFABP.**
(TIF)

Figure S4 **Structure plots for (A) K = 2 and (B) K = 3.**
(TIF)

Table S1 **Individual counts of** ***Hemidactylus frenatus*** **,** ***Lepidodactylus lugubris*** **, and** ***Gehyra oceanica*** **in 7 buildings in Moorea.** Localities: 1) Gump Station; 2) Magasin Ami René; 3) Herman Perles in Hauru; 4) Magasin Lai-Assan in Haaptit; 5) Chez Teima in Maatea; 6) Electricite de Tahiti in Vaiare; 7) Magasin Lee Hen in Pao Pao.
(DOC)

Table S2 **Diversity at each of the nuclear loci.** Based on Tamura-Nei corrected average pairwise divergence (*D*a; above diagonal); within-population θ_π_ (diagonal); population pairwise *F*_ST_ based on Tamura & Nei genetic differences (below diagonal). Bold numbers indicate statistically significant (p<0.05).
(DOC)

Table S3 **Sequences sampled with associated Genbank accession numbers.**
(DOC)
