# Supplementary figures and images for: High Genetic Diversity Despite the Potential for Stepping-Stone Colonizations in an Invasive Species of Gecko on Moorea, French Polynesia
Source: PLoS One. 2011 Nov 2;6(11):e26874. doi: 10.1371/journal.pone.0026874 (PMC3206873; doi:10.1371/journal.pone.0026874)

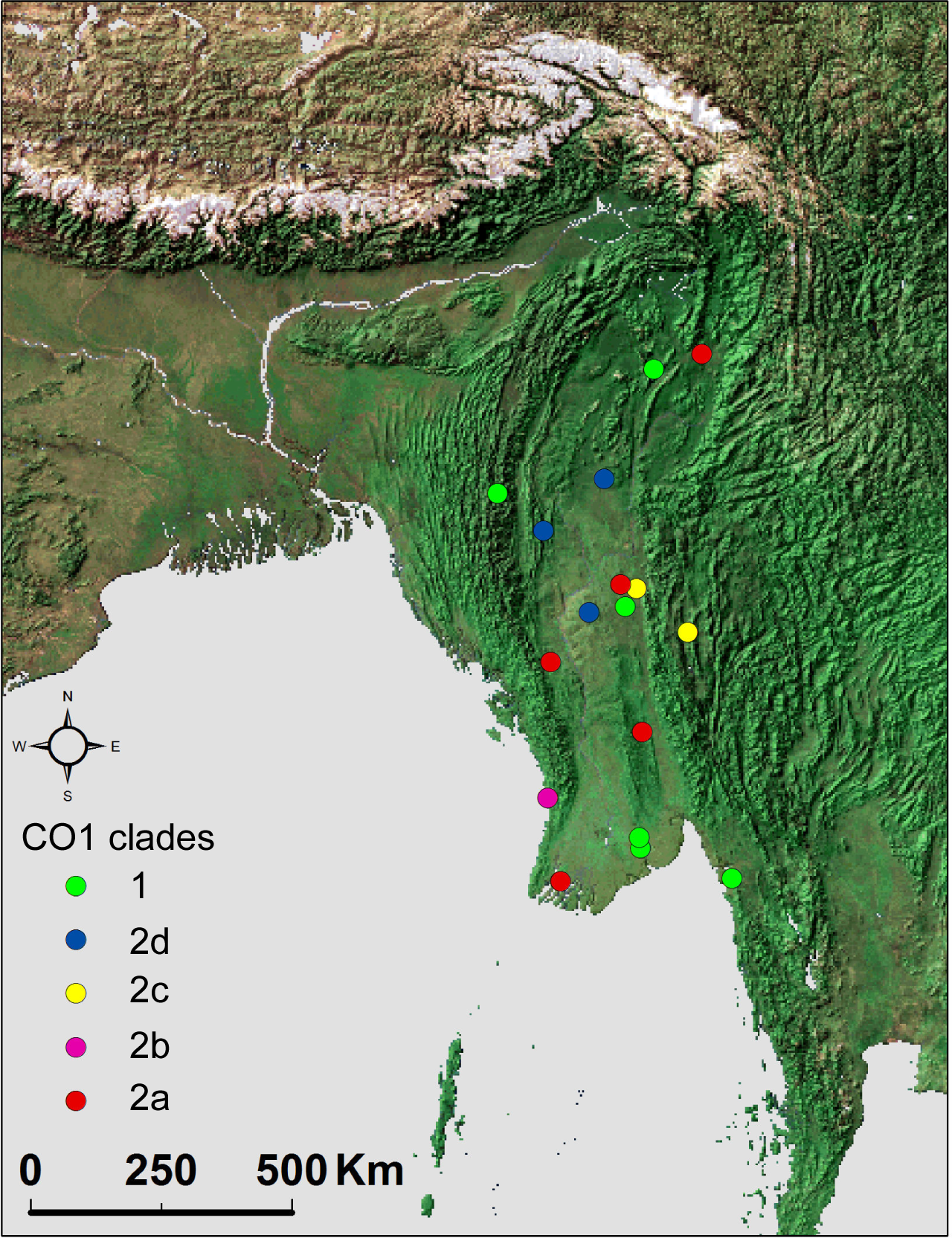

Supplement: Figure S1 — Map of Myanmar showing location of major CO1 lineages. (TIF) [file pone.0026874.s001.tif]

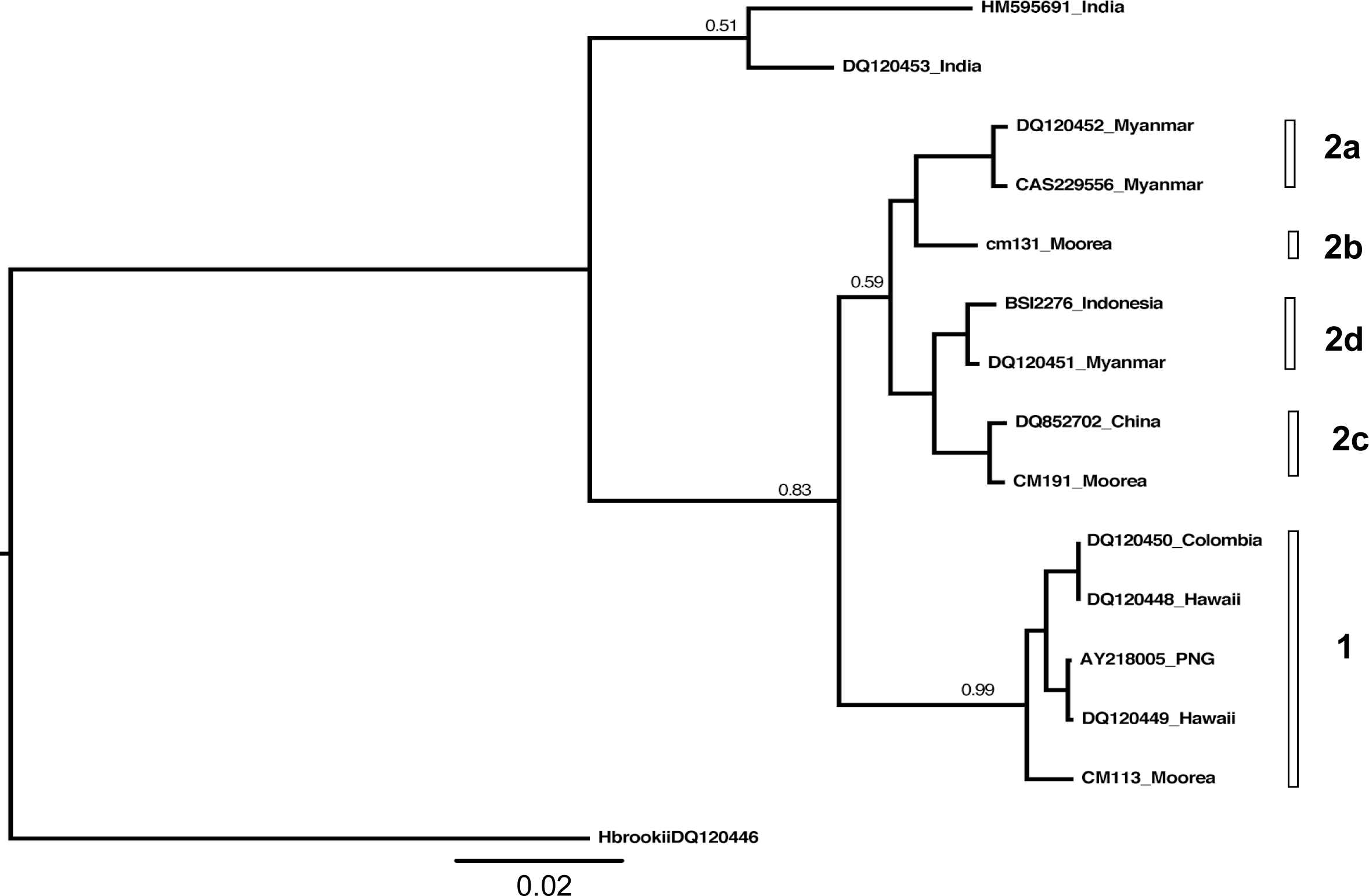

Supplement: Figure S2 — Bayesian phylogeny of 12S sequences. Neighbor joining tree includes 12S sequences from this study, Bansal and Karanth [29], Carranza and Arnold [33], Feng et al. [35], and Whiting et al. [34]. Numbers to the right of the names represent major CO1 groups found in this study. Posterior probabilities for the major groups are included on the branches. (TIF) [file pone.0026874.s002.tif]

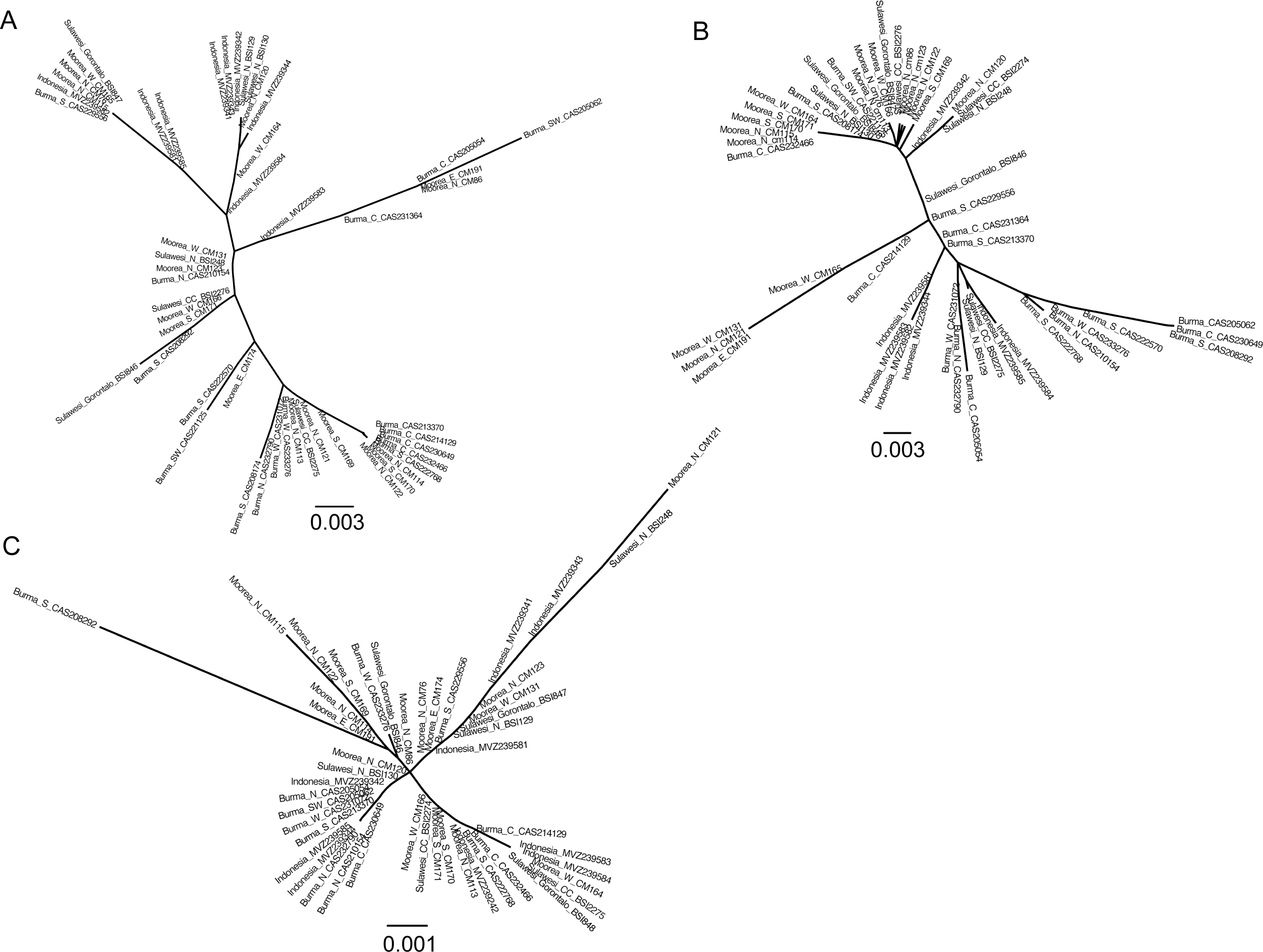

Supplement: Figure S3 — Unrooted Neighbor-joining network of (A) rpl18, (B) rpl14, and (C) LFABP. (TIF) [file pone.0026874.s003.tif]

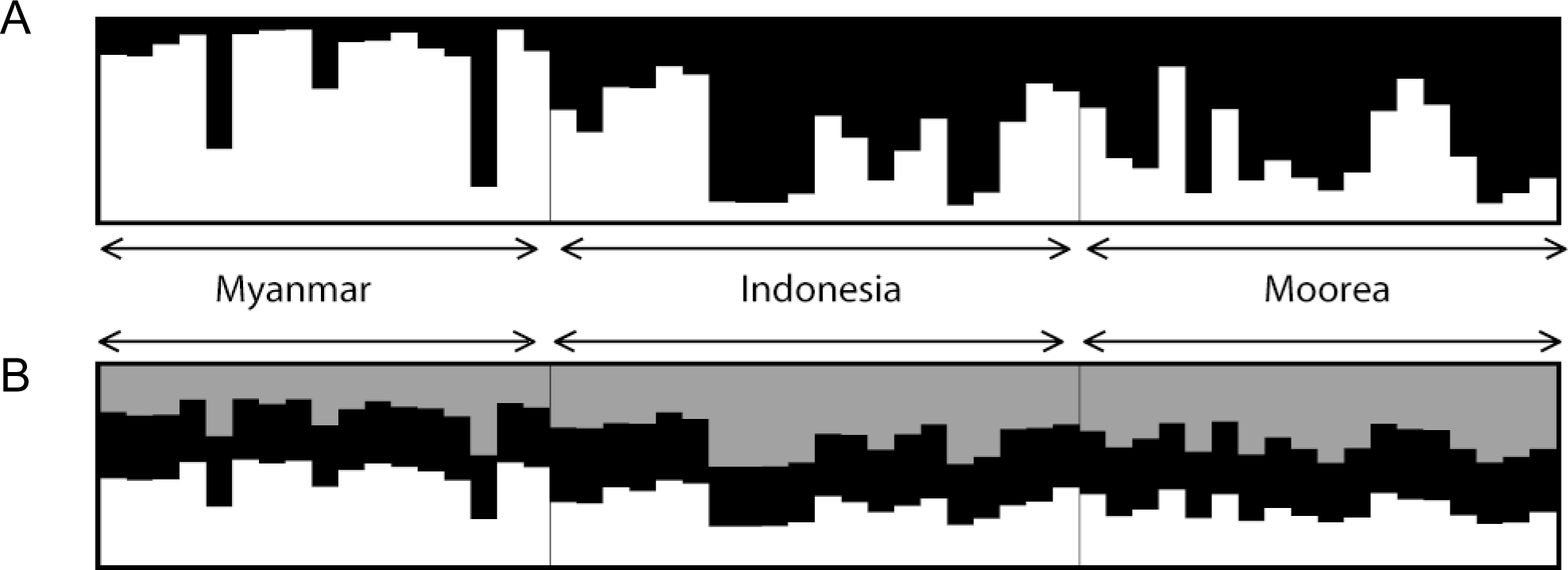

Supplement: Figure S4 — Structure plots for (A) K = 2 and (B) K = 3. (TIF) [file pone.0026874.s004.tif]
