# Supplementary material for: High Genetic Diversity Despite the Potential for Stepping-Stone Colonizations in an Invasive Species of Gecko on Moorea, French Polynesia
Source: PLoS One. 2011 Nov 2;6(11):e26874. doi: 10.1371/journal.pone.0026874 (PMC3206873; doi:10.1371/journal.pone.0026874)
Supplement: Table S1 — Individual counts of Hemidactylus frenatus , Lepidodactylus lugubris , and Gehyra oceanica in 7 buildings in Moorea. Localities: 1) Gump Station; 2) Magasin Ami René; 3) Herman Perles in Hauru; 4) Magasin Lai-Assan in Haaptit; 5) Chez Teima in Maatea; 6) Electricite de Tahiti in Vaiare; 7) Magasin Lee Hen in Pao Pao. (DOC) [file pone.0026874.s005.doc]

.

| Locality | *H. frenatus* | *L. lugubris* | *G. oceanica* |
| --- | --- | --- | --- |
| 1 | 6 | 1 | 0 |
| 2 | 13 | 0 | 0 |
| 3 | 7 | 1 | 0 |
| 4 | 6 | 0 | 1 |
| 5 | 24 | 0 | 0 |
| 6 | 7 | 0 | 0 |
| 7 | 3 | 1 | 0 |
