# Supplementary material for: High Genetic Diversity Despite the Potential for Stepping-Stone Colonizations in an Invasive Species of Gecko on Moorea, French Polynesia
Source: PLoS One. 2011 Nov 2;6(11):e26874. doi: 10.1371/journal.pone.0026874 (PMC3206873; doi:10.1371/journal.pone.0026874)
Supplement: Table S2 — Diversity at each of the nuclear loci. Based on Tamura-Nei corrected average pairwise divergence (Da; above diagonal); within-population θπ (diagonal); population pairwise F ST based on Tamura & Nei genetic differences (below diagonal). Bold numbers indicate statistically significant (p<0.05). (DOC) [file pone.0026874.s006.doc]

|  | LFABP |  |  |  | rpl14 |  |  |  | rpl18 |  |  |  |
| --- | --- | --- | --- | --- | --- | --- | --- | --- | --- | --- | --- | --- |
|  | n | Moorea | Indonesia | Myanmar | n | Moorea | Indonesia | Myanmar | n | Moorea | Indonesia | Myanmar |
| Moorea | 17 | 0.0097 | 0.0000 | 0.0009 | 17 | 0.0193 | 0.0053 | 0.0128 | 18 | 0.0199 | 0.0013 | 0.0013 |
| Indonesia | 17 | 0.0736 | 0.0083 | 0.0006 | 16 | 0.2156 | 0.0195 | 0.0056 | 16 | 0.0643 | 0.0173 | 0.0057 |
| Myanmar | 13 | 0.0983 | -0.0047 | 0.0064 | 17 | 0.3293 | 0.1760 | 0.0330 | 17 | 0.0621 | 0.2409 | 0.0186 |
