# Supplementary material for: High Genetic Diversity Despite the Potential for Stepping-Stone Colonizations in an Invasive Species of Gecko on Moorea, French Polynesia
Source: PLoS One. 2011 Nov 2;6(11):e26874. doi: 10.1371/journal.pone.0026874 (PMC3206873; doi:10.1371/journal.pone.0026874)
Supplement: Table S3 — Sequences sampled with associated Genbank accession numbers. (DOC) [file pone.0026874.s007.doc]

|  | | | | GenBank Accession numbers | | | | |
| --- | --- | --- | --- | --- | --- | --- | --- | --- |
| Specimen Voucher | Field ID | CO1 group | Locality | CO1 | *12s* | *rpl14* | *rpl18* | *LFABP* |
| MVZ239242 |  | 5 | Indonesia | JN705609 |  | JN705455 |  | JN705553 |
| MVZ239341 |  | 5 | Indonesia | JN705630 |  |  |  | JN705551 |
| MVZ239342 |  | 5 | Indonesia | JN705607 |  |  |  | JN705591 |
| MVZ239343 |  | 5 | Indonesia | JN705597 |  |  |  | JN705566 |
| MVZ239344 |  | 5 | Indonesia | JN705635 |  | JN705440 |  | JN705557 |
| MVZ239581 |  | 5 | Indonesia | JN705638 |  | JN705446 |  | JN705592 |
| MVZ239582 |  | 5 | Indonesia | JN705600 |  | JN705439 |  |  |
| MVZ239583 |  | 5 | Indonesia | JN705616 |  | JN705456 |  | JN705573 |
| MVZ239584 |  | 5 | Indonesia | JN705648 |  | JN705457 |  | JN705562 |
| MVZ239585 |  | 5 | Indonesia | JN705617 |  | JN705472 |  | JN705568 |
| BSI846 |  | 3 | Indonesia | JN705643 |  | JN705470 | JN801228 | JN705588 |
| BSI847 |  | 5 | Indonesia | JN705620 |  | JN705481 | JN801200 | JN705546 |
| BSI848 |  | 3 | Indonesia | JN705629 |  | JN705454 |  | JN705567 |
| BSI129 |  | 5 | Indonesia | JN705613 |  | JN705487 | JN801235 | JN705559 |
| BSI130 |  | 5 | Indonesia | JN705642 |  | JN705453 | JN801238 | JN705579 |
| BSI248 |  | 5 | Indonesia | JN705640 |  | JN705471 | JN801213 | JN705569 |
| BSI2274 |  | 5 | Indonesia | JN705634 |  | JN705477 |  | JN705589 |
| BSI2275 |  | 5 | Indonesia | JN705621 |  | JN705450 | JN801224 | JN705564 |
| BSI2276 |  | 3 | Indonesia | JN705605 | JN700063 | JN705444 | JN801233 |  |
| MVZ266344 | CM113 | 5 | Moorea | JN705623 | JN700062 | JN705460 | JN801202 | JN705556 |
| MVZ266345 | CM114 | 4 | Moorea | JN705612 |  | JN705461 | JN801199 | JN705576 |
| MVZ266346 | CM115 | 5 | Moorea | JN705650 |  | JN705479 | JN801234 | JN705571 |
| MVZ266350 | CM120 | 5 | Moorea | JN705594 |  | JN705442 | JN801189 | JN705582 |
| MVZ266351 | CM121 | 5 | Moorea | JN705608 |  | JN705445 | JN801198 | JN705587 |
| MVZ266352 | CM122 |  | Moorea | JN705593 |  | JN705469 | JN801220 | JN705570 |
| MVZ266353 | CM123 | 5 | Moorea | JN705601 |  | JN705459 | JN801197 | JN705574 |
| MVZ266349 | CM131 | 1 | Moorea | JN705625 | JN700059 | JN705452 | JN801208 | JN705554 |
| MVZ266348 | CM76 | 4 | Moorea | JN705596 |  | JN705449 | JN801201 | JN705586 |
| MVZ266347 | CM86 | 4 | Moorea | JN705645 |  | JN705463 | JN801227 | JN705555 |
| MVZ266432 | CM165 |  | Moorea |  |  | JN705451 | JN801230 |  |
| MVZ266433 | CM166 | 5 | Moorea | JN705604 |  | JN705464 | JN801221 | JN705565 |
| MVZ266435 | CM169 | 4 | Moorea | JN705644 |  | JN705483 |  | JN705561 |
| MVZ266436 | CM170 |  | Moorea |  |  | JN705438 | JN801226 | JN705548 |
| MVZ266437 | CM171 |  | Moorea | JN705599 |  | JN705484 | JN801210 | JN705560 |
| MVZ266438 | CM174 |  | Moorea |  |  |  | JN801203 | JN705563 |
| MVZ266431 | CM164 | 5 | Moorea | JN705624 |  | JN705480 | JN801211 | JN705583 |
| MVZ266434 | CM191 | 4 | Moorea | JN705637 | JN700060 | JN705485 | JN801195 | JN705578 |
| MVZ266509 | Toni10 | 4 | Moorea | JN705647 |  |  |  |  |
| MVZ266505 | Toni15 | 4 | Moorea | JN705614 |  |  |  |  |
| MVZ266506 | Toni16 | 5 | Moorea | JN705611 |  |  |  |  |
| MVZ266507 | Toni18 | 5 | Moorea | JN705622 |  |  |  |  |
| MVZ266508 | Toni30 | 5 | Moorea | JN705595 |  |  |  |  |
| MVZ266511 | Toni45 | 4 | Moorea | JN705619 |  |  |  |  |
| MVZ266510 | Toni9 | 4 | Moorea | JN705606 |  |  |  |  |
| CAS205054 |  | 4 | Myanmar | JN705598 |  | JN705462 | JN801216 | JN705585 |
| CAS205062 |  | 1 | Myanmar | JN705628 |  | JN705465 | JN801196 | JN705577 |
| CAS208174 |  | 5 | Myanmar | JN705610 |  | JN705476 | JN801219 |  |
| CAS208292 |  | 2 | Myanmar | JN705603 |  | JN705447 | JN801222 | JN705584 |
| CAS210154 |  | 3 | Myanmar | JN705649 |  | JN705468 | JN801218 | JN705547 |
| CAS213370 |  | 5 | Myanmar | JN705618 |  | JN705467 | JN801206 | JN705550 |
| CAS214129 |  | 3 | Myanmar | JN705626 |  | JN705466 | JN801212 | JN705572 |
| CAS221125 |  | 5 | Myanmar | JN705636 |  | JN705443 | JN801229 |  |
| CAS222570 |  | 5 | Myanmar | JN705631 |  | JN705458 | JN801190 |  |
| CAS222768 |  | 2 | Myanmar | JN705633 |  | JN705448 | JN801192 | JN705549 |
| CAS229556 |  | 2 | Myanmar | JN705639 | JN700061 | JN705441 | JN801191 | JN705580 |
| CAS230649 |  | 4 | Myanmar | JN705632 |  | JN705482 | JN801237 | JN705552 |
| CAS231072 |  | 2 | Myanmar | JN705615 |  | JN705474 | JN801223 | JN705581 |
| CAS231364 |  | 2 | Myanmar | JN705627 |  | JN705475 | JN801204 |  |
| CAS232466 |  | 3 | Myanmar | JN705646 |  | JN705478 | JN801214 | JN705558 |
| CAS232790 |  | 5 | Myanmar | JN705641 |  | JN705473 | JN801193 | JN705575 |
| CAS233276 |  | 5 | Myanmar | JN705602 |  | JN705486 | JN801232 | JN705590 |
